# Nucleus accumbens pathways control cell-specific gene expression in the medial prefrontal cortex

**DOI:** 10.1038/s41598-020-58711-2

**Published:** 2020-02-04

**Authors:** Takatoshi Hikida, Shuhei Yao, Tom Macpherson, Ayumi Fukakusa, Makiko Morita, Haruhide Kimura, Keisuke Hirai, Tatsuya Ando, Hiroyoshi Toyoshiba, Akira Sawa

**Affiliations:** 1 Laboratory for Advanced Brain Functions, Institute for Protein Research, Osaka University, 3-2 Yamadaoka, Suita, Osaka 565-0871 Japan; 2 Department of Research and Drug Discovery, Medical Innovation Center, Kyoto University Graduate School of Medicine, 53 Shogoin-Kawahara-cho, Sakyo-ku, Kyoto 606-8507 Japan; 3 Research, Takeda Pharmaceutical Company Limited, 2-26-1 Muraoka-Higashi, Fujisawa, Kanagawa 251-8555 Japan; 4 Departments of Psychiatry, Johns Hopkins University School of Medicine, Baltimore, MD 21287 USA; 5 Departments of Neuroscience, Johns Hopkins University School of Medicine, Baltimore, MD 21287 USA; 6 Departments of Biomedical Engineering, Johns Hopkins University School of Medicine, Baltimore, MD 21287 USA; 7 Departments of Genetic Medicine, Johns Hopkins University School of Medicine, Baltimore, MD 21287 USA; 8 Department of Mental Health, Johns Hopkins University Bloomberg School of Medicine, Baltimore, MD 21287 USA

**Keywords:** Neural circuits, Molecular neuroscience

## Abstract

The medial prefrontal cortex (mPFC) is a critical component of a cortico-basal ganglia-thalamo-cortical loop regulating limbic and cognitive functions. Within this circuit, two distinct nucleus accumbens (NAc) output neuron types, dopamine D1 or D2 receptor-expressing neurons, dynamically control the flow of information through basal ganglia nuclei that eventually project back to the mPFC to complete the loop. Thus, chronic dysfunction of the NAc may result in mPFC transcriptomal changes, which in turn contribute to disease conditions associated with the mPFC and basal ganglia. Here, we used RNA sequencing to analyse differentially expressed genes (DEGs) in the mPFC following a reversible neurotransmission blocking technique in D1 or D2 receptor-expressing NAc neurons, respectively (D1-RNB, or D2-RNB). Gene Set Enrichment Analysis revealed that gene sets of layer 5b and 6 pyramidal neurons were enriched in DEGs of the mPFC downregulated in both NAc D1- and D2-RNB mice. In contrast, gene sets of layer 5a pyramidal neurons were enriched in upregulated DEGs of the mPFC in D1-RNB mice, and downregulated DEGs of the mPFC in D2-RNB mice. These findings reveal for the first time that NAc output pathways play an important role in controlling mPFC gene expression.

## Introduction

Cortico-basal ganglia-thalamo-cortical circuits are known to play critical roles in motor control, decision-making, and cognitive functions, and, as such, their dysfunction is proposed to underlie various movement and psychiatric conditions^1–5^. While motor control is thought to be largely regulated through a sensorimotor loop involving the motor cortices and the dorsal striatum, a loop containing the medial prefrontal cortex (mPFC) and the nucleus accumbens (NAc) of the ventral striatum has been identified in the control of several limbic and cognitive functions^6^. Accordingly, alterations in mPFC neural networks have been suggested to play a considerable role in disorders associated with cognitive impairments, including schizophrenia^7^.

The NAc is a major target of mPFC efferent projections, and activity in separate populations of NAc dopamine D1 or D2 receptor-expressing GABAergic medium spiny neurons (D1-/D2-MSNs) is implicated in reward and aversion learning, respectively^8–12^. While NAc D2-MSNs project exclusively to the ventral pallidum (VP), D1-MSNs project to both the substancia nigra pars reticulata (SNr) and the VP^13^. GABAergic VP neurons project to the SNr, which in turn sends inhibitory efferents to the medial dorsal and ventral dorsal thalamus (MD/VD)^14,15^. Finally, the MD and VD send glutamatergic efferents back to the mPFC to complete the loop. Thus, signalling in NAc D1- and D2-MSNs is likely to be able to alter activity in the mPFC, which may in turn regulate mPFC inputs into the basal ganglia, as well as other regions, in order to modulate decision-making and behaviour. Indeed, in the parallel cortico-basal ganglia-thalamo-cortical sensorimotor loop, it has been shown that optogenetic activation of dorsal striatal D1- and D2-MSNs is able to enhance or suppress, respectively, the activity of primary motor cortex neurons^15,16^. This connectivity between the NAc and the mPFC raises the possibility that chronic dysregulation of NAc activity could produce long-lasting alterations in mPFC gene regulation, contributing to the development of disorders associated with dysfunction of the mPFC-basal ganglia-thalamo-mPFC circuit.

Here we used a TetTag method to induce reversible neurotransmission blocking (RNB) specifically in NAc D1- or D2-MSNs by combining the use of a neurotransmission-blocking tetanus toxin (TeNT) transgenic mouse line with intra-NAc infusions of an adeno-associated virus that drives the expression of TeNT exclusively in either D1- or D2-expressing cells in the NAc. Following chronic blockade of neurotransmission in NAc D1- or D2-MSNs using RNB, we performed RNA sequencing (RNA-seq) to evaluate changes at the transcriptome level in the mPFC using two major analysis methods, edgeR and limma’s voom function (voom), which calculate differential expression of genes based upon negative binomial distributions or empirical Bayes estimates of RNA-seq counts, respectively. Subsequently, Ingenuity Pathway Analysis was used to investigate whether differentially expressed genes (DEGs) in the mPFC following NAc D1-D2-RNB are associated with disease-related functions. Finally, a Gene Set Enrichment Analysis (GSEA) computational method was used to determine whether *a priori* defined sets of genes, identified from to be expressed in specific CNS cell lines by Translating Ribosome Affinity Purification (TRAP), were significantly enriched or depleted by NAc D1-D2-RNB compared to the equivalent sets in control mice (see methods for further details)^17–19^.

Our findings reveal a significant role for NAc D1- and D2-MSN pathways in controlling gene expression within the mPFC, and indicate that chronic inhibition of NAc signalling results in altered expression of genes associated with movement, sleep, developmental, and psychiatric disorders. Finally, transmission blocking of NAc D1- or D2-MSNs bidirectionally altered gene expression in genes associated with Layer 5a pyramidal neurons, known to project to striatal regions^19,20^, suggesting that chronic dysfunction of distinct NAc output pathways results in transcriptomal changes that may affect specific cortical pathways within the mPFC-basal ganglia-thalamo-mPFC circuit.

## Results

### EdgeR and voom gene expression analysis of the mPFC after NAc D1-MSN or D2-MSN pathway inhibition

**Figure 1:**
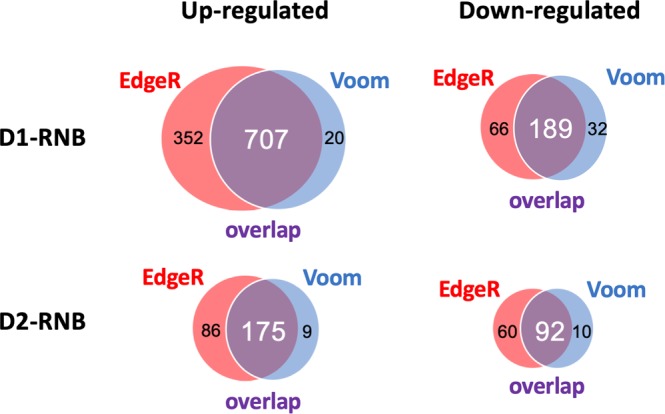
Comparison of differentially expressed genes (DEGs) in the mPFC by edgeR/voom. Venn diagrams show the number of up-regulated (left) and down-regulated (right) DEGs in the mPFC of D1-RNB (top) and D2-RNB (bottom) mice calculated individually by edgeR (red circles) and voom (blue circles) methods. DEGs identified by both methods are shown in overlapped (purple) circles.

Two methods, edgeR and voom, were applied to gene expression data and DEGs were individually identified. After comparing mPFC samples from D1-RNB mice with those from WT controls, edgeR and voom identified DEGs were significantly overlapped for 707 up-regulated genes (Fig. 1; −logP = 2093.32) and 189 down-regulated genes (Fig. 1; −logP = 780.94). Whereas, in the mPFC samples of D2-RNB mice compared with WT controls, edgeR and voom identified DEGs were significantly overlapped for 175 up-regulated genes (Fig. 1; −logP = 753.31) and 92 down-regulated genes (Fig. 1; −logP = 427.43). Of these DEGs identified by both edgeR and voom methods, only 25 up-regulated genes (−logP = 15.59) and 9 down-regulated genes (−logP = 15.32) were shared by both D1-RNB and D2-RNB mice. Only one gene, Gpr88, was observed to be down-regulated in NAc D1-RNB mice and up-regulated in NAc D2-RNB mice, and is known to be a modulator of the striatal dopamine system^21^. Genes identified by both edgeR and voom in D1- or D2-RNB mice were defined as robust DEGs and were used for subsequent hierarchical clustering and Ingenuity Pathway Analysis (IPA).

After normalization (see Methods), hierarchical clustering of the expression profile of 1163 robust DEGs in the mPFC was performed to investigate whether gene expression patterns were similar within genotypes and different between genotypes. With the exception of one D1-RNB sample, the expression profiles for all 1163 DEGs were clustered into three groups corresponding to each genotype (x axis in Supplementary Fig. S1). Additionally, within these groups, four distinct clusters were identified, corresponding to the groups of DEGs up-regulated or down regulated in D1-RNB and D2-RNB mice (y axis in Supplementary Fig. S1). These findings indicate distinct intra-region expression differences in the mPFC between wildtype, NAc D1-RNB, and NAc D2-RNB mice.

### Enrichment of gene sets related to neurological diseases in the mPFC of NAc D1-RNB and D2-RNB mice

To evaluate whether robust DEGs in the mPFC are associated with disease-related functions, functional enrichment analysis by IPA was performed. Down-regulated DEGs in the mPFC of D1-RNB mice were found to be significantly overlapped with gene sets functionally related with disorders associated with impaired motor control, including “dyskinesia” (*p* = 1.96E-03), “Huntington’s disease” (*p* = 2.52E-03), and “neuromuscular disorder” (*p* = 2.61E-03), as well as with “primary insomnia” (*p* = 7.14E-04), and developmental disorders associated with impaired cognition, including “autism” (*p* = 1.58E-03) and “attention deficit hyperactivity disorder” (*p* = 2.25E-03) (Table 1). Up-regulated DEGs in the mPFC of D2-RNB mice were similarly found to be overlapped with gene sets related to several disorders associated with impaired motor control, including “Movement disorders” (*p* = 5.63E-06), “neuromuscular disease” (*p* = 2.65E-05), “Huntington’s disease” (*p* = 1.19E-05), and “catalepsy” (*p* = 1.79E-04), as well as with gene sets functionally associated with “sleep disorders” (*p* = 1.59E-04) and “cocaine-related disorder” (*p* = 3.30E-06) (Table 2). These findings confirm that chronic inhibition of signalling in NAc D1- and D2-MSNs alters mPFC expression of genes associated with disorders of the mPFC-basal ganglia-thalamo-mPFC circuit.

**Table 1:** Top 10 functions associated with down-regulated genes in the medial prefrontal following D1-RNB.

Function	p-value	Genes	Number of genes
Primary insomnia	7.14E-04	GABRA5, GABRB2, HTR2A	3
Epileptic encephalopathy	1.32E-03	GABRA5, GABRB2, HCN1, HTR2A, NECAP1	5
Neurological signs	1.47E-03	ATP6V1A, CETN2, CHN1, CRYM, FBXW7, FDFT1, FLRT2, GABRA5, GABRB2, GPR88, GUCY1B3, HSP90AA1, HSPA8, HTR2A, MME, NPTX2, OPRM1, SGTB	18
Autism	1.58E-03	ALDH1A3, CADPS2, GABRA5, GABRB2, HTR2A	5
Dyskinesia	1.96E-03	ATP6V1A, CETN2, CHN1, CRYM, FBXW7, FDFT1, FLRT2, GABRA5, GABRB2, GPR88, GUCY1B3, HSP90AA1, HSPA8, HTR2A, NPTX2, OPRM1, SGTB	17
Ohtahara syndrome	2.08E-03	GABRA5, HCN1, HTR2A, NECAP1	4
Attention deficit hyperactivity disorder	2.25E-03	GABRA5, GABRB2, HTR2A, OPRM1	4
Huntington’s Disease	2.52E-03	ATP6V1A, CETN2, CHN1, CRYM, FBXW7, FDFT1, FLRT2, GABRA5, GABRB2, GPR88, GUCY1B3, HSP90AA1, HSPA8, HTR2A, OPRM1, SGTB	16
Seizure disorder	2.60E-03	CD83, CHN1, CRYM, ELAVL4, GABRA5, GABRB2, HCN1, HSPA8, HTR2A, NECAP1, NPTX2, OPRM1, SH3GL2, SV2B	14
Neuromuscular disease	2.61E-03	ATP6V1A, CD55, CETN2, CHN1, CRYM, FBXW7, FDFT1, FLRT2, GABRA5, GABRB2, GPR88, GUCY1B3, HSP90AA1, HSPA8, HTR2A, IFIT1, KCNA3, MDH1, OPRM1, SGTB, SH3GL2	21

**Table 2:** Top 10 functions associated with up-regulated genes in the medial prefrontal following D2-RNB.

Function	p-value	Genes	Number of genes
Disorders of the basal ganglia	1.29E-06	ADORA2A, AP1S2, CPT1B, DRD1, DRD2, DYNLT1, GPR6, GPR88, GRK1, IDO1, IL4, LPL, NUDT1, PDE4C, PPP1R1B, PTGES3, RASGRP2, RGS9, SCN9A, SLC18A2, SPOCK3, SYNPR, TSC22D3	23
Cocaine-related disorder	3.30E-06	ADORA2A, DRD1, DRD2, ESR1, PDE4C, SLC18A2	6
Movement Disorders	5.63E-06	ABCB7, ADORA2A, AP1S2, COMMD3-BMI1, CPT1B, DRD1, DRD2, DYNLT1, EFHC1, GNB4, GPR6, GPR88, GRK1, IDO1, IL4, LPL, NUDT1, PDE4C, PPP1R1B, PTGES3, RASGRP2, RGS9, SCN9A, SLC18A2, SPOCK3, SYNPR, TSC22D3	27
Huntington’s Disease	1.19E-05	ADORA2A, CPT1B, DRD1, DRD2, DYNLT1, GPR6, GPR88, GRK1, IDO1, LPL, PPP1R1B, RASGRP2, RGS9, SCN9A, SLC18A2, SPOCK3, SYNPR, TSC22D3	18
Neuromuscular disease	2.65E-05	ADORA2A, AP1S2, CPT1B, DRD1, DRD2, DYNLT1, GPR6, GPR88, GRK1, IDO1, IL4, LPL, PDE4C, PPP1R1B, PTGES3, RASGRP2, RGS9, SCN9A, SLC18A2, SPOCK3, SYNPR, TSC22D3	22
Advanced idiopathic Parkinson disease	7.71E-05	DRD1, DRD2	2
Idiopathic Parkinson disease	1.05E-04	ADORA2A, DRD1, DRD2	3
Sleep Disorders	1.59E-04	ADORA2A, DRD1, DRD2, ESR1, GLRA2, PDE4C	6
Catalepsy	1.79E-04	ADORA2A, DRD2, PPP1R1B	3
Huntington disease grade 0 Huntington’s disease	2.30E-04	ADORA2A, DRD2	2

### Estimation of CNS cell types altered by NAc D1-RNB and D2-RNB

**Figure 2:**
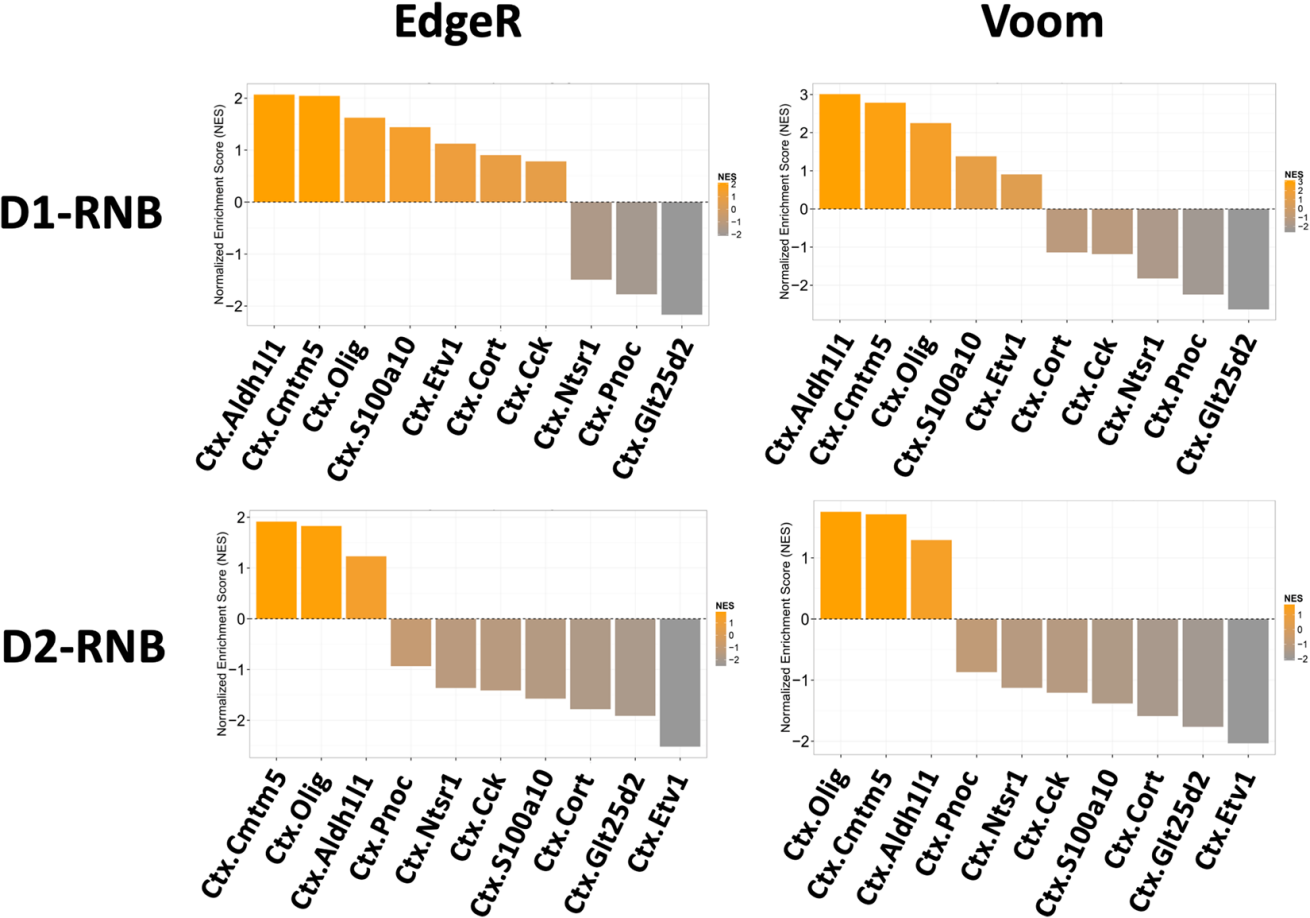
GSEA estimation of altered cortex cell types in the mPFC by NAc D1-RNB/D2-RNB. Barplots indicate the GSEA-based normalized enrichment scores (NES) of cortical cell gene sets calculated by edgeR (left two panels) or voom (right two panels) for NAc D1-RNB vs WT (top two panels) and NAc D2-RNB vs WT (bottom two panels). X-axis: cortical cell types ordered by NES. Y-axis: NES are also displayed by colour gradient. Ctx.Aldh1l1; cortical astrocytes, Ctx.Cck; Cortical Cck+ neurons, Ctx.Cmtm5; cortical mature oligodendrocytes, Ctx.Cort; cortical Cort+ neurons, Ctx.Etv1; layer 5a pyramidal neurons, Ctx.Glt25d2; layer 5b pyramidal neurons, Ctx.Ntsr1; layer 6 pyramidal neurons, Ctx.Olig; cortical mixed oligodendroglia, Ctx.Pnoc; cortical Pnoc+ neurons, Ctx.S100a10; layer 5a pyramidal neurons.

The GSEA computational method was used to calculate the degree to which the expression patterns of *a priori* defined gene sets for specific cell types were altered by chronic neurotransmission blocking of NAc D1- or D2-MSNs. GSEA using gene sets identified from TRAP data from 25 CNS cell types (Table 3) was performed separately on the full datasets for edgeR and voom to investigate potential cell types in the mPFC contributing to differences between NAc D1-RNB and D2-RNB. Of 10 cortical cell types, gene sets for mature oligodendrocytes (Ctx.Cmtm5) (Fig. 2; D1-RNB; edgeR *p* < 1.0E-03, voom *p* < 1.0E-03, D2-RNB; edgeR *p* < 1.0E-03, voom *p* < 1.0E-03), mixed oligodendroglia (Ctx.Olig) (Fig. 2; D1-RNB; edgeR *p* < 1.0E-03, voom *p* < 1.0E-03, D2-RNB; edgeR *p* = 6.7E-03, voom: *p* = 3.6E-03), and astrocytes (Ctx.AldhL1) (Fig. 2; D1-RNB; edgeR *p* < 1.0E-03, voom *p* < 1.0E-03, D2-RNB; edgeR *p* = 0.19, voom *p* = 0.081) were found to be significantly enriched in genes up-regulated in the mPFC of both D1- and D2-RNB mice, indicating that chronic blockade of neurotransmission in NAc D1- and D2-MSN pathways results in increased expression genes associated with several types of glial cells within the mPFC.

**Table 3:** Description of gene sets for 25 cell types analysed by GSEA.

Gene Set Name	Brain region	Description	Source (GSE No.)
Bf.Chat	Basal Forebrain	Cholinergic Neurons	GSE13379
Bs.Chat	Brainstem	Motor Neurons	GSE13379
Cb.Aldh1l1	Cerebellum	Astrocytes	GSE13379
Cb.Cmtm5	Cerebellum	Mature Oligodendrocytes	GSE13379
Cb.Grm2	Cerebellum	Golgi Neurons	GSE13379
Cb.Grp	Cerebellum	Unipolar Brush Neurons	GSE13379
Cb.Lypd6	Cerebellum	Stellate and Basket Neurons	GSE13379
Cb.Neurod1	Cerebellum	Granule Neurons	GSE13379
Cb.Olig	Cerebellum	Mixed Oligodendroglia	GSE13379
Cb.Pcp2	Cerebellum	Purkinje Neuron	GSE13379
Cb.Sept4	Cerebellum	Bergmann Glia	GSE13379
Cs.Chat	Striatum	Cholinergic Neurons	GSE13379
Cs.Drd1	Striatum	Drd1+ Medium Spiny Neurons	GSE13379
Cs.Drd2	Striatum	Drd2+ Medium Spiny Neurons	GSE13379
Ctx.Aldh1l1	Cortex	Astrocytes	GSE13379
Ctx.Cck	Cortex	Cck+ Neurons	GSE13379
Ctx.Cmtm5	Cortex	Mature Oligodendrocytes	GSE13379
Ctx.Cort	Cortex	Cort+ Neurons	GSE13379
Ctx.Etv1	Cortex	Layer 5a Pyramidal Neurons	GSE35766
Ctx.Glt25d2	Cortex	Layer 5b Pyramidal Neurons	GSE35766
Ctx.Ntsr1	Cortex	Layer 6 Pyramidal Neurons	GSE35766
Ctx.Olig	Cortex	Mixed Oligodendroglia	GSE13379
Ctx.Pnoc	Cortex	Pnoc+ Neurons	GSE13379
Ctx.S100a10	Cortex	Layer 5a Pyramidal Neurons	GSE35766
Sc.Chat	Spinal Cord	Cholinergic Neurons	GSE13379

Of cortical neuron gene sets, gene sets for Glt25d2- (Ctx.Glt25d2) and Ntsr1-expressing (Ctx.Ntsr1) neurons, previously identified to be layer 5b and layer 6 pyramidal neurons, respectively^20,22^, were enriched in genes down-regulated by both NAc D1-RNB (Fig. 2; Ctx.Glt25d2: edgeR *p* = 9.3E-03, voom *p* < 1.0E-03, Ctx.Ntsr1: edgeR *p* = 1.1E-02, voom *p* = 0.01) and NAc D2-RNB (Fig. 2; Ctx.Glt25d2: edgeR *p* < 1.0E-03, voom: *p* < 1.0E-03, Ctx.Ntsr1: edgeR *p* = 2.9E-03, voom *p* = 2.2E-03). Additionally, gene sets for cell types previously identified to be layer 5a pyramidal neurons^19,20^ were revealed to be enriched in genes up-regulated in the mPFC of NAc D1-RNB mice (Ctx.S100a10 gene set only) (Fig. 2; Ctx.S100a10: edgeR: *p* = 3.1E-03, voom: *p* = 0.013) and down-regulated in the mPFC of D2-RNB mice (both Ctx.S100a10 and Ctx.Etv1 gene sets) (Fig. 2: Ctx.S100a10: edgeR: *p* < 1.0E-03, voom: *p* = 2.1E-03, Ctx.Etv1: edgeR *p* = 3.2E-03, voom *p* < 1.0E-03). Thus, chronic neurotransmission blocking in NAc D1- or D2-MSN pathways reduces the expression of both layer 5b and Layer 6 pyramidal neuron-related genes, but increases or decreases, respectively, the expression of layer 5a pyramidal neuron-related genes. The complete set of results for the 25 cell types are shown in Supplementary Fig. S2.

### S100a10-expressing layer 5a pyramidal neuron-related gene expression differs in the mPFC of NAc D1-RNB and NAc D2-RNB mice

**Figure 3:**
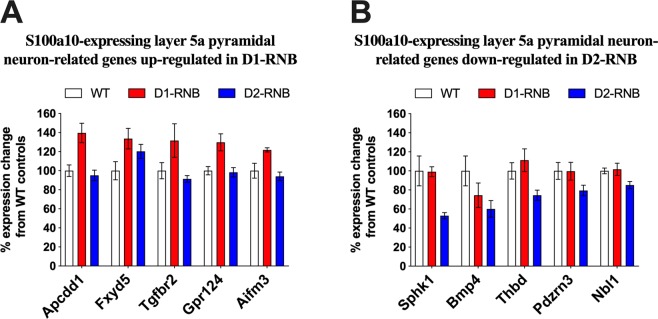
S100a10-expressing layer 5a pyramidal neuron-related genes up- and down-regulated in the mPFC by D1-RNB and D2-RNB, respectively. (**A**) Percentage expression change from WT controls of the five most up-regulated genes in the S100a10-expressing layer 5a pyramidal neuron gene set in the mPFC of NAc D1-RNB mice, alongside WT and D2-RNB mice, as identified by voom. (**B**) Percentage expression change from WT controls of the five most down-regulated genes in the S100a10-expressing layer 5a pyramidal neuron gene set in the mPFC of NAc D2-RNB mice, alongside WT and D1-RNB mice, as identified by voom. Bars represent the mean percentage expression change from WTs ±SEM.

Given that gene set for S100a10-expressing layer 5a pyramidal neurons (Ctx.S100a10) was enriched in both genes up-regulated in the mPFC of D1-RNB mice and down-regulated in the mPFC of D2-RNB mice, we investigated whether opposing patterns of expression were occurring in the same genes. The five most up-regulated genes in the Ctx.S100a10 gene set in mPFC samples of NAc D1-RNB mice (Fig. 3A) were found to differ from the five most down-regulated genes in the gene set in mPFC samples of NAc D2-RNB mice (Fig. 3B). Thus, while chronic neurotransmission blocking of NAc D1- and D2-MSN produced a general bidirectional effect on the expression of genes within this layer 5a pyramidal neuron gene set, the degree to which the expression of individual genes within this gene set was bidirectionally controlled differed.

## Discussion

**Figure 4:**
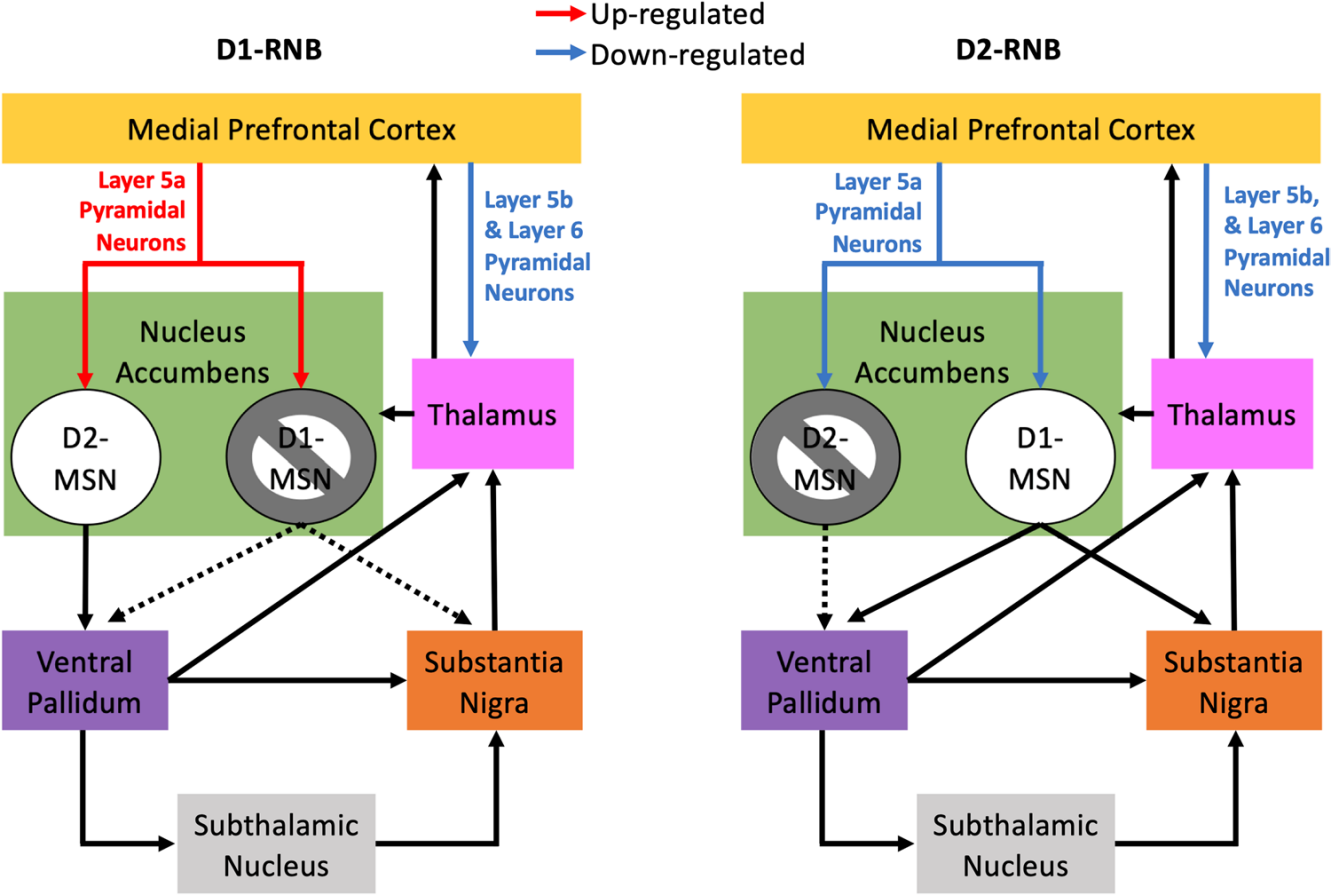
Schematic of changes in cortical neurons following reversible neurotransmission blocking in NAc D1- or D2-MSNs. Gene sets for layer 5b and 6 pyramidal neurons (Ctx.Glt5d2, Ctx.Ntsr1) were enriched in genes down-regulated by NAc D1- and D2-RNB. The gene sets for layer 5a pyramidal neurons (Ctx.S100a10 and Ctx.Etv1) was enriched in genes up-regulated in NAc D1-RNB mice and down-regulated in NAc D2-RNB mice.

The mPFC and NAc form critical components of an mPFC-basal ganglia-thalamo-mPFC loop circuit that is proposed to link cognition to action selection, and dysfunction of this circuit has been suggested to contribute to several neuropathologies associated with impaired cognitive function and behavioural control^1,2,5,7,11^. Here, for the first time, we reveal that blocking neurotransmission in NAc D1- or D2-MSNs was able to alter the mPFC transcriptome, producing different patterns of gene expression within specific cell types in the mPFC (see Fig. 4).

Up-regulated or down-regulated genes in the mPFC showed little overlap between D1- and D2-RNB groups, and gene expression profiles could be hierarchically clustered into three groups corresponding to each genotype. This would suggest that similar to their distinct roles in controlling various forms of learning and behaviour, NAc D1- and D2-MSNs also have largely divergent influences on gene expression within the mPFC. Further investigation using ingenuity pathway analysis revealed that mPFC genes down-regulated by NAc D1-RNB and up-regulated by NAc D2-RNB significantly overlapped with several gene sets functionally associated with developmental and psychiatric disorders characterized by impaired cognition, as well as with gene sets for movement-related disorders. Many of these overlapped genes were present in multiple gene sets and were responsible for diverse and important roles throughout the brain, including encoding for several types of receptors, enzymes, and monoamine transporters. However, it is still unclear whether transcriptomal changes in the mPFC result in altered expression of proteins, and further research is warranted to investigate whether potential translational alterations contribute to functional impairments associated with chronic inhibition of signalling in NAc D1- and D2-MSNs, including behavioural and learning dysfunctions^8,10,11,23^.

GSEA using gene sets for cortical cell types revealed that chronic blockade of neurotransmission in NAc D1- and D2-MSN pathways results in increased mPFC expression of genes associated with several types of glial cells, including mature oligodendrocytes, mixed oligodendroglia, and astrocytes. Given the diverse functions of glial cells, the enrichment of these gene sets may produce a considerable influence on mPFC function, and further investigation of the consequences of gene expression up-regulation in glial cells is necessary.

Of cortical neuron groups, gene expression in gene sets for layer 5b and 6 pyramidal neuron-types was found to be significantly down-regulated in the mPFC of both D1-RNB and D2-RNB mice. Previous studies using BAC Cre-recombinase driver lines to study these neuron types have revealed that Gltd25d2-expressing layer 5b pyramidal neurons project to areas of the lateral dorsal and lateral posterior thalamic nuclei, the striatum, and the pontine nuclei^22^, while Ntsr1-expressing layer 6 pyramidal neurons project largely to the dorsal thalamic nuclei^20^. Interestingly, the gene sets for layer 5a pyramidal neurons were revealed to be bidirectionally enriched in the mPFC following blockade of neurotransmission in NAc pathways, with NAc D1-RNB resulting in increased expression of genes in the gene set for S100a10-expressing neurons, and NAc D2-RNB resulting in decreased expression of genes in the gene set for S100a10- and Etv1-expressing neurons. Similar tracing studies using Cre-recombinase driver lines have demonstrated these layer 5a pyramidal neurons project to both the NAc and the dorsomedial striatum (DMS)^19,20^.

mPFC layer 5a, 5b, and 6 pyramidal neurons are known to play critical roles in controlling learning and decision-making processes. Thalamic projections from mPFC layer 6 neurons (“modulator” efferents) and, to a lesser extent, layer 5b neurons (“driver” efferents conveying processed cortical information) have been suggested to act as a part of a higher-order thalamic relay projecting information back to cortical areas including the mPFC to facilitate learning^24^. Whereas, DMS-projecting mPFC corticostriatal neurons have recently been shown to act in parallel with sensorimotor corticostriatal neurons projecting from the motor cortex M1 to the dorsolateral striatum (DLS) to control the early stages of motor skill learning, but are disengaged during and after mastery of the skill, at which time M1-DLS corticostriatal neuron activity plays an important role in solidifying the behaviour^25^. These findings led to the suggestion that mPFC-DMS corticostriatal neurons control attention and act to promote behavioural flexibility during early learning when multiple strategies are being tested. Recent evidence that inactivation of either mPFC corticostriatal or corticothalamic neurons impairs performance in reversal learning tasks further highlights the importance of mPFC neurons in adaptively controlling learning^26,27^. Interestingly, our previous work has demonstrated that NAc D2-RNB mice show similar impairments in reversal learning tasks, suggesting that a cortico-basal ganglia-thalamo-cortical circuit incorporating NAc D2-MSNs may play an important role in the ability for behavioural flexibility^23,28^.

While here we identify several cell types in which gene expression was specifically altered, a limitation of the current study is that the functional consequences of these transcriptional changes are still unclear. Future studies using *in-vivo* imaging or electrophysiological techniques in BAC cre-recombinase driver lines will likely provide valuable information about how these transcriptional changes alter signalling within specific cortical neurons during tasks that typically engage these projection neurons, including reward-based and reversal learning tasks. If discovered, these cortical neuron signalling alterations may help to explain deficits in reward, aversive, and reversal learning tasks previously reported in NAc D1- and D2-RNB mice^8,10,23,28^. Additionally, further studies combining the use of BAC transgenic mouse lines expressing cre-recombinase in specific cell-types with cre-specific anterograde and retrograde viruses expressing fluorescent markers, will help confirm the efferent projections of several of the cell types described in this study, as well as their possible presence within cortico-basal ganglia-thalamo-cortical circuits.

Finally, a further use for these data could be to screen the location of action and mechanisms of new drugs that act to alter signalling within the mPFC-basal ganglia-thalamo-cortical circuit, as well as to compare against gene expression profiling of small molecule treatments for the purpose of repurposing existing drugs that may have undiscovered effects on gene regulation. For example, drugs resulting in up-regulation of genes in the layer 5a pyramidal neuron gene set in the mPFC may be producing their action by decreasing signalling within NAc D1-MSNs. Similarly, in future, the gene expression patterns of specific mPFC cells types in NAc D1- and D2-RNB mice reported here could be compared with gene expression patterns in the same mPFC cell types of mouse models of psychiatric disorders associated with impaired mPFC function to help to identify how specific mPFC-basal ganglia-thalamo-mPFC pathways are altered in these conditions.

In conclusion, here, for the first time, we demonstrate that inhibition of NAc D1- and D2-MSNs produces similar or opposing changes in gene expression within specific mPFC cell types, and identify important mPFC cellular targets for future research into the role of NAc pathways in controlling the function of the mPFC-basal ganglia-thalamo-mPFC circuit (see Fig. 4**)**. Further research is warranted to establish the functional influence of these transcriptomal changes on signalling within these mPFC cell types, and the extent to which these expression changes contribute to altered learning and behaviour reported in NAc D1- and D2-RNB mice.

## Materials and Methods

### Animal care

All recombinant mouse and viral experiment procedures were approved by the Institutional Safety Committee on Recombinant DNA Experiments (approval ID 04219), Animal Experimental Committee of Institute for Protein Research at Osaka University (approval ID 29-02-1), the Animal Care and Use Committee of Kyoto University (approval ID MedKyo17071). These procedures were performed under the institutional guidelines. Mice were housed in a temperature-controlled room at 22 °C with a 12 h light/dark cycle. Fresh water and rodent diet were available *ad libium*.

### Reversible neurotransmission blocking

A reversible neurotransmission blocking (RNB) technique was used to inhibit signalling in NAc D1- or D2-MSNs (D1-/D2-RNB), according to a previously described method^8–10,23^. Briefly, transgenic mice in which tetanus toxin light chain (TN) is expressed under the control of tetracycline-responsive element (TRE) received intra-NAc infusions one of two recombinant AAV viruses that express tetracycline transactivator (tTA) under a promotor for either Substance P (expressed in D1-MSNs) or Enkephalin (expressed in D2-MSNs) (AAV2-SP-tTA or AAV2-Enk-tTA; 500 nl/site at 100 nl/min; left for 5 min; coordinates: AP +1.4, L ±0.8 and DV −4.0 and −3.5). The interaction between the SP/ENK-expressed tTA and the TRE drives the release of neurotransmission blocking TN specifically in NAc D1- (SP) or D2- (Enk) MSNs, and is only reversed if doxycycline is administered. In the case of this study, mice were left with a permanent blockade of neurotransmission within NAc D1- or D2-MSNs.

### RNA extraction and sequencing

Following cervical dislocation, freshly dissected brains were sliced into 1 mm-thick coronal sections using a mouse brain matrix. The medial prefrontal cortex (mPFC), identified using mouse brain atlas^29^, was dissected from brain slices using a 1.5 mm-diameter tissue puncher and frozen at −80 °C until the time of use. Total RNA were extracted from four mPFC samples from wild type (WT) mice, three mPFC samples from D1-RNB mice, and three mPFC samples from D2-RNB mice using the miRNeasy Mini Kit (QIAGEN, Hilden, Germany). The quality of purified RNA was assessed with an Experion^TM^ Automated Electrophoresis Station using the Experion^TM^ RNA StdSens Analysis Kit for mPFC (Bio-Rad Laboratories, CA, U.S.A.). RNA sequencing (RNA-seq) was conducted using Illumina HiSeq. 2000 in Beijing Genomics Institute (Hong Kong, China).

### Statistical analysis of RNA-seq data

RNA-seq data were processed using Array Studio 8.0.0.78 (OmicSoft Corp., Cary, NC, U.S.A.) as follows. FASTQ files were mapped using mouse.mm10 as the reference library, and then gene annotations were added to the read count values with ensembl.R76. After checking the mapping rate was over 85%, the read counts of all samples were exported. Statistical analysis was conducted by R language (https://www.r-project.org/). To identify mRNAs differentially expressed in the mPFC of D1-/D2-RNB and WT mice, the edgeR (empirical analysis of DGE in R) package^30^ and the voom (an acronym of “variance modeling at the observational level”) function in the limma package^31^ were applied. These are two major methods for calculation of RNA-Seq data set. To get robust differentially expressed genes, intersected genes by edgeR and voom were selected. For both edgeR and voom, only genes with a cpm value of more than 1 in at least one sample were kept for further analysis. Up- and down-regulated genes were identified by a p-value of less than 0.05 and a fold change of more than 1.2. Percentage expression change from WT controls for up-regulated and down-regulated genes was analysed using the averaged cpm for each group divided by the averaged cpm for the WT group and multiplied by 100. The top five up-regulated Ctx.S100a10-associated genes by D1-RNB and top five down-regulated Ctx.S100a10-associated genes by D2-RNB were analysed by univariate ANOVAs with the percentage expression change from WT controls as the dependent variable and genotype as the independent variable. Clustering of differentially expressed genes (DEGs) was conducted by spearman’s correlation and an average method with R. A hypergeometric function was used to perform the enrichment analysis to compare DEGs by each method.

### Enrichment analysis by ingenuity pathway analysis

The function of DEGs was investigated using Ingenuity Pathway Analysis (QIAGEN, Hilden, Germany) applied with a filter to capture only enriched gene sets related to “neurological diseases”, with the background gene set assigned to genes with a cpm > 1 for at least one comparison group.

### Identification of CNS cell type-specific gene sets

A Specificity Index (SI) was used to identify gene sets specifically expressed in 25 CNS cell types (Table 3) isolated using TRAP (from Gene Expression Omnibus GSE13379 and GSE35766 data sets^18,19^). In the SI method, CEL files were processed by RMA using the Affy package. After normalization by quantile methods and transformation to log2 values, probes with values ≥ 25 percentile in at least 1 sample were extracted. To determine highly expressed genes in the isolated cells but not in the surrounding tissues, q-values < 0.05 and log2ratio > −1 were applied as cut-offs for Welch’s t-test. Using the SI calculated from the gene lists obtained by t-tests, gene lists for the enrichment analysis were produced using *p* < 0.01 against the data set.

### Gene set enrichment analysis

Gene Set Enrichment Analysis (GSEA) was performed using java implementation GSEA software downloaded from the Broad Institute (http://www.broadinstitute.org/gsea) according to a previously described method^17^. Briefly, a normalized enrichment score (NES) is calculated for each gene set based upon the degree to which genes within the set are overrepresented at the extremes of a list of genes ranked by expression. Here, ranked gene lists as a query for GSEA were created separately from edgeR/voom data from D1-RNB/D2-RNB vs WT comparisons, rather than from the intersect list of “robust DEGs”, due to the requirement of the full gene expression data set for GSEA.

## Supplementary information

Supplementary information.

## Data Availability

All RNA-seq data sets generated and/or analysed during the current study were added to the Gene Expression Omnibus (GEO) under the accession number GSE124583.
