# Supplementary material for: Nucleus accumbens pathways control cell-specific gene expression in the medial prefrontal cortex
Source: Sci Rep. 2020 Feb 4;10:1838. doi: 10.1038/s41598-020-58711-2 (PMC7000772; doi:10.1038/s41598-020-58711-2)
Supplement: Supplementary file 1 — Supplementary information. [file 41598_2020_58711_MOESM1_ESM.docx]

**Supplemental Figures**

**Nucleus accumbens pathways control cell-specific gene expression**

**in the medial prefrontal cortex**

Takatoshi Hikida, Shuhei Yao, Tom Macpherson, Ayumi Fukakusa, Makiko Morita, Haruhide Kimura, Keisuke Hirai, Tatsuya Ando, Hiroyoshi Toyoshiba, Akira Sawa

**
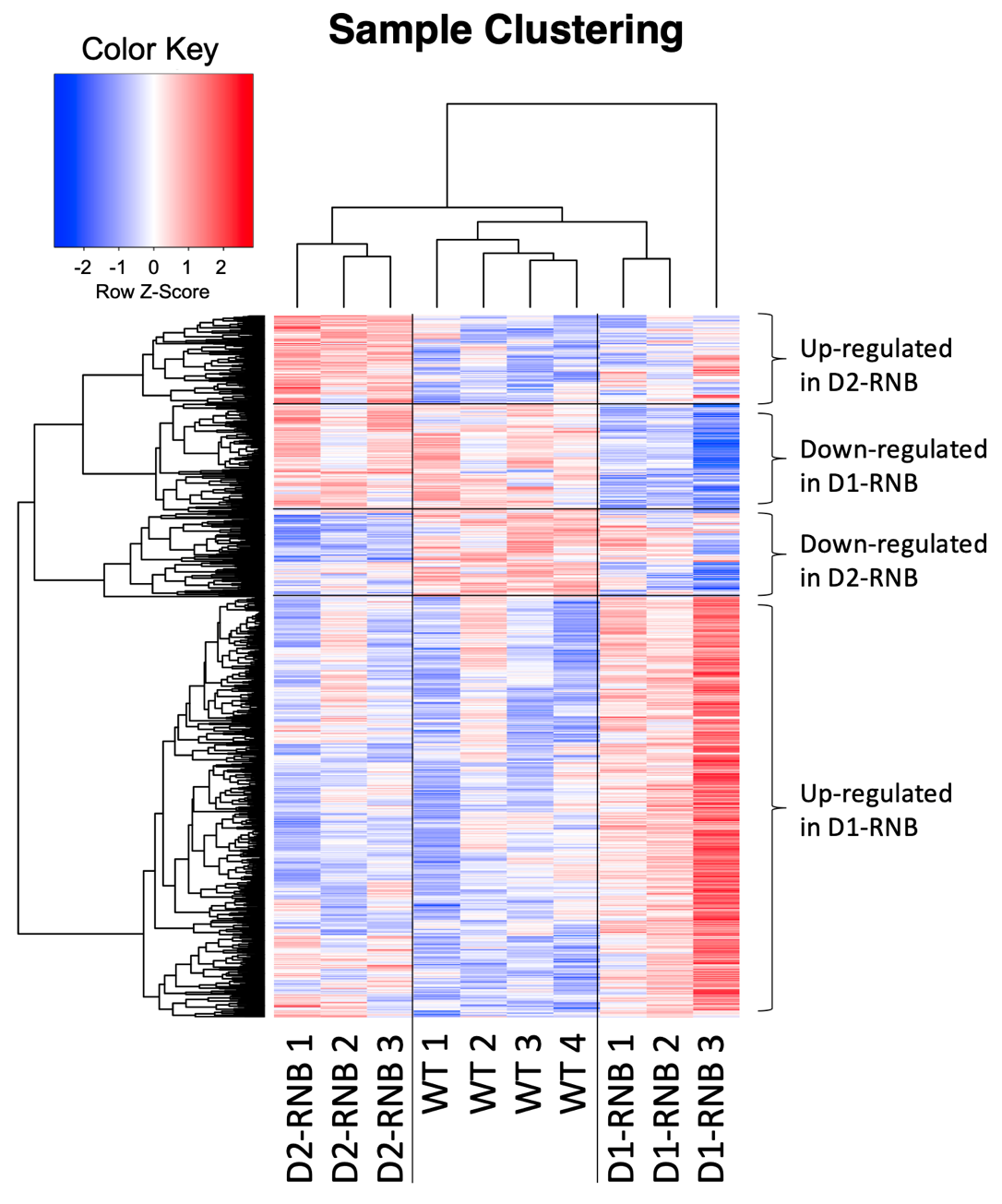
**

**Supplementary Figure S1: Heatmap of hierarchical clustering of gene expression values of 1163 DEGs overlapped between edgeR and voom following NAc D1- and D2-RNB**. D1-RNB arrays showed clusters of concordant gene expression patterns that differed from those of D2-RNB and WT controls. The colour key demonstrates the extent to which gene expression values differ from the row mean (row z-score) in units of standard deviations, with red indicating up-regulated expression and blue indicating down-regulated expression.

**
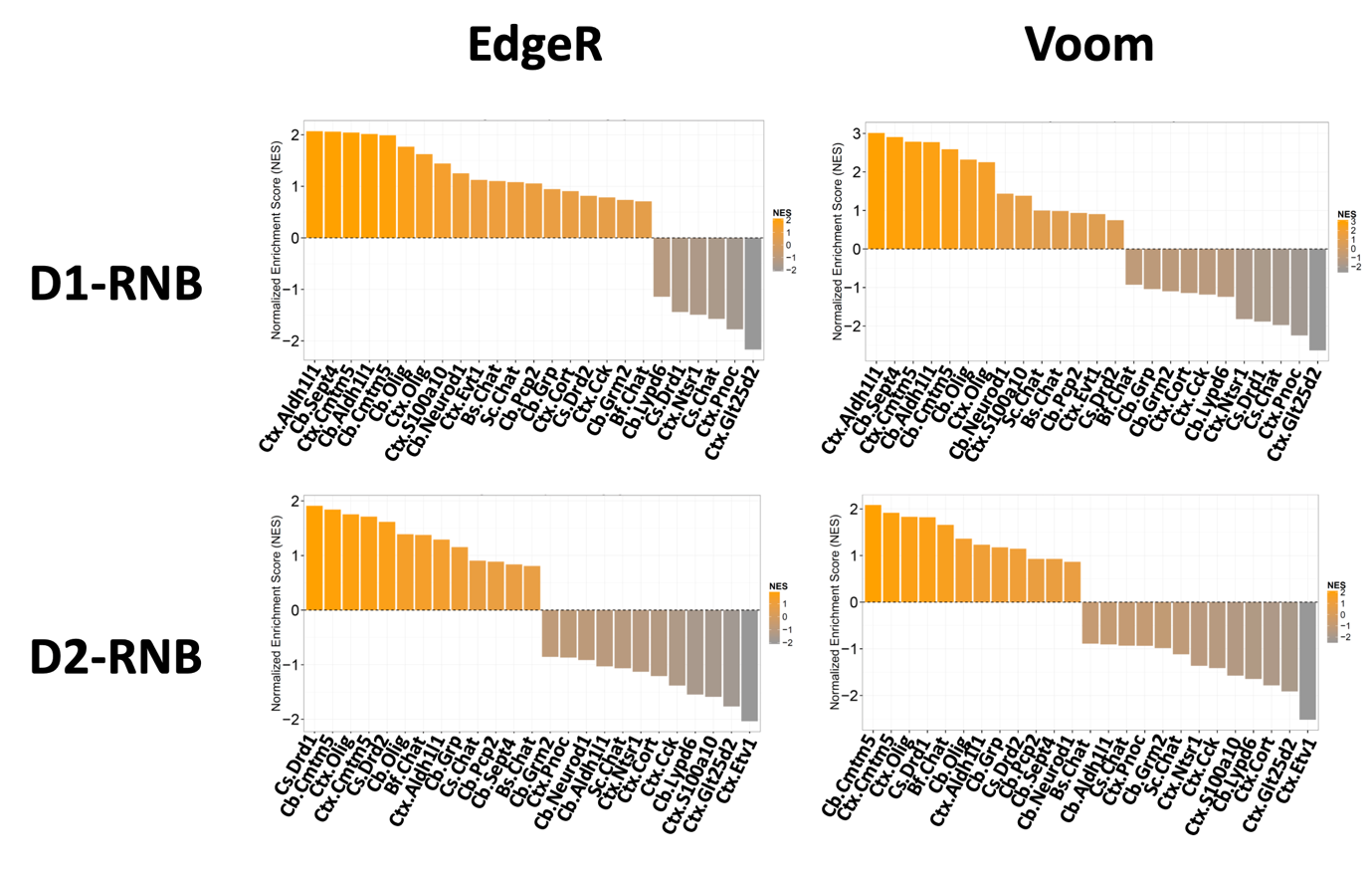
**

**Supplementary Figure S2: GSEA estimation of altered cell types by NAc D1-RNB / D2-RNB using data from 25 cell types.** Barplots indicate GSEA-based normalized enrichment scores (NES) of gene sets for 25 cell types calculated by edgeR (left two panels) or voom (right two panels) for NAc D1-RNB vs WT (top two panels) and NAc D2-RNB vs WT (bottom two panels). X-axis: 25 cell types ordered by NES. Y-axis: NES are also displayed by colour gradient. Bf.Chat; basal forebrain cholinergic neurons, Bs.Chat; brainstem cholinergic neurons, Cb.Aldh1l1; cerebellar astrocytes, Cb.Cmtm5; cerebellar mature oligodendrocytes, Cb.Grm2; cerebellar golgi neurons, Cb.Grp; cerebellar unipolar brush neurons, Cb.Lypd6; cerebellar stellate and basket neurons, Cb.Neurod1; cerebellar granule neurons, Cb.Olig; cerebellar mixed oligodendroglia, Cb.Pcp2; cerebellar Purkinje neurons, Cb.Sept4; cerebellar Bergmann glia, Cs.Chat; striatal cholinergic neurons, Cs.Drd1; striatal Drd1+ medium spiny neurons, Cs.Drd2; striatal Drd2+ medium spiny neurons, Ctx.Aldh1l1; cortical astrocytes, Ctx.Cck; Cortical Cck+ neurons, Ctx.Cmtm5; cortical mature oligodendrocytes, Ctx.Cort; cortical Cort+ neurons, Ctx.Etv1; layer 5a pyramidal neurons, Ctx.Glt25d2; layer 5b pyramidal neurons, Ctx.Ntsr1; layer 6 pyramidal neurons, Ctx.Olig; cortical mixed oligodendroglia, Ctx.Pnoc; cortical Pnoc+ neurons, Ctx.S100a10; layer 5a pyramidal neurons, Sc.Chat; spinal cord cholinergic neurons.
